# Central retinal artery occlusion as a first sign of atrial fibrillation: A 3‐year retrospective single‐center analysis

**DOI:** 10.1002/clc.23673

**Published:** 2021-10-28

**Authors:** Nadine Vonderlin, Karsten Kortuem, Johannes Siebermair, Martin Köhrmann, Tienush Rassaf, Steffen Massberg, Siegfried Priglinger, Stefan Kääb, Reza Wakili

**Affiliations:** ^1^ Department of Cardiology and Vascular Medicine, West‐German Heart and Vascular Center Essen University of Essen Medical School, University Duisburg‐Essen Essen Germany; ^2^ Medical Clinic I University of Munich Munich Germany; ^3^ German Centre for Cardiovascular Research (DZHK) Berlin Germany; ^4^ Eye Hospital University of Munich Munich Germany; ^5^ Department of Neurology University of Essen Medical School, University Duisburg‐Essen Essen Germany

**Keywords:** (C)RAO, AF, anticoagulation, stroke

## Abstract

**Background:**

Central retinal artery occlusion ((C)RAO) is known to be associated with stroke and/or atrial fibrillation (AF). Nevertheless, patients often present at the ophthalmologist initially and it is unknown how many of these receive an adequate cardiological/neurological work‐up (CWU/NWU), including a 24 h‐Holter‐ECG.

**Hypothesis:**

Hypothesis of this study was that patients with (C)RAO do not undergo CWU on regular basis and that new‐onset AF is more often detected in patients with CWU.

**Methods and results:**

We performed a retrospective analysis of *n* = 292 consecutive patients who presented at an ophthalmology department with the diagnosis of (C)RAO during a 3‐year period. After excluding patients with known AF, meeting exclusion criteria, inability to comply with the protocol, missed land phoneline, or death during follow‐up a total of 174 patients were enrolled; mean follow‐up was 20 ± 12 months. The CHA_2_DS_2_‐VASc score of the cohort was 5.3 ± 1.4. Our analysis revealed that only 50.6% of patients received a CWU including a single Holter‐ECG after the index‐event. In 12.6% cases new‐onset AF was diagnosed, while the rate was higher in patients with CWU compared to patients without CWU (18.2 vs. 7.0%; *p* = 0.26). Evaluation of oral anticoagulation (OAC) therapy showed that only 66% of patients with AF were treated according to guidelines.

**Conclusion:**

Only half of patients with (C)RAO underwent CWU. Despite minimal monitoring, rate of new diagnosed AF was high. Our results confirm that (C)RAO identifies a high‐risk population for AF. These results illustrate the importance to implement standardized CWU in (C)RAO patients presenting at the ophthalmologist.

## 1 BACKGROUND

Embolic events, that is, stroke, associated with atrial fibrillation (AF) have important relevance for patients' prognosis and socioeconomic burden. In recent years, the issue of AF detection in context of stroke underwent a great change and received a lot of attention. The CRYSTAL AF trial was the first trial to investigate systematically patients with cryptogenic stroke and to screen for silent AF. Patients, which were continuously monitored, showed a higher incidence of AF compared to patients, who received a routine work‐up with 24 h‐Holter‐ECG.^1^ Based on these findings studies were initiated to evaluate the potential benefit of an oral anticoagulation for prevention of recurrent stroke in patients after an ESUS (embolic stroke of unknown source).^2^, ^3^, ^4^

Another disease type with a close pathophysiological similarity is the (central) retinal artery occlusion ((C)RAO), which could be considered as a stroke equivalent. (C)RAO has an incidence between 1 and 15/10.000 und can be classified in thromboembolic, non‐arteritic (ca. 95%), or arteritic (ca. 5%) occlusion.^5^ The mean age of affected patients is 60 to 65 years and the majority of patients (>90%) are older than 40 years.^6^, ^7^ Men are more often affected than women (2/3 to 1/3).^8^

Patients who suffer from (C)RAO present with acute and painless reduction or even loss of monocular vision. The main time for presenting at an ophthalmologist is ~23 h.^9^ Reasons for such a long waiting period are mainly misjudgment and the poor knowledge of this disease in the general population.^10^ Furthermore, the initial presentation is mainly performed at an ophthalmologist due to loss of vision. This frequently prevents a timely neurological or cardiological diagnostic work‐up regarding the thromboembolic source. However, there is no therapeutic approach. Intra‐arterial fibrinolysis warrants in CRAO no outcome improvement.^11^ As another consequence, the required diagnostic work‐up evaluating central atherosclerotic disease or the presence of AF is not regularly performed, prohibiting a potential required prevention therapy. With respect to the underlying causes of (C)RAO atherosclerosis of ipsilateral carotid artery is one of the two main causes. Depending on studies, the prevalence of significant stenosis of brain supplying arteries differs ranging from 10% to 70%.^12^, ^13^, ^14^, ^15^ The second important cause is cardiogenic embolism with a prevalence ranging up to 48%.^14^ Commonly associated risk factors of (C)RAO are arterial hypertension, atrial fibrillation, coronary heart disease as well as hypercholesterinemia, diabetes mellitus, and smoking which further underline the fact that we are dealing with a systemic condition.^14^, ^16^

Registry studies showed that after experiencing a (C)RAO event the risk of subsequent stroke is increased 2–4 times within 3 years.^17^, ^18^ These numbers highlight that AF, stroke and (C)RAO are related to each other and that a cardiovascular/neurological diagnostic work‐up is justified and recommended by current guidelines.^19^

## 2 HYPOTHESIS

Objective of this study was to investigate the number of patients admitted to a ophthalmology department with the diagnosis of (C)RAO, who received further cardiological/neurological work‐up regarding potential AF diagnosis or carotid disease screening identifying the underlying pathophysiological cause. In addition, we aimed to evaluate in how many patients new‐onset AF was detected by a regular cardiological routine work‐ and follow‐up in comparison to patients who did not undergo any cardiological work‐up or specific rhythm monitoring.

## 3 METHODS

### 3.1 Patient enrollment

This retrospective monocentric (LMU University Hospital, Munich) study enrolled all patients, who presented with the diagnosis of (C)RAO at the department of ophthalmology in the period between 01/2014 and 11/2016. They were identified by using ophthalmic hospital's ''Smart eye Database''.^20^ Inclusion criteria were documented diagnosis of (C)RAO, age ≥ 18 and ≤ 80 and given informed consent. Main exclusion criteria were documented history of atrial fibrillation, inability to comply study protocol, missed land phonelines as well as decease during follow up.

### 3.2 Predefined endpoints

The primary endpoint was the percentage of patients who had undergone a cardiological work‐up (CWU), which was defined as receiving at least one Holter‐ECG for ≥ 24 h after the index (C)RAO event.

A key secondary endpoint was the number of patients who were diagnosed with de‐novo AF during the follow‐up period defined as documented AF according to the current definitions^21^ ‐ either in standard‐ or Holter‐ECG ‐ in the corresponding groups (patients without CWU vs. with CWU). All ECGs were collected and evaluated by two independent physicians blinded to the respective diagnostic arm.

Other secondary endpoints comprised the number of patients who received CWU in an ambulatory versus clinical setting, patients who underwent a neurological work‐up (NWU; defined as a duplex ultrasound of brain suppling arteries), number of patients with severe carotid stenosis (NASCET criteria, defined as ≥70% stenosis) and evaluation of antithrombotic therapy (no therapy, antiplatelet therapy, or anticoagulation). Another secondary analysis aimed to investigate the number of patients who experienced a recurrent thromboembolic event (stroke or (C)RAO) during the follow‐up period. Diagnosis was based on the patient's statement or provided discharge letter if available. To evaluate the potential risk for stroke, for each patient a CHA_2_DS_2_VASc‐Score equivalent was calculated as if atrial fibrillation would be present.

### 3.3 Follow‐up

After obtaining informed consent for retrospective evaluation, we investigated the patient follow‐up since the index event and used a standardized questionnaire concerning follow‐up examinations (CWU and NWU), cardiovascular risk factors (arterial hypertension, diabetes mellitus), antiplatelet or anticoagulation therapy, if the patient had already a stroke or (C)RAO prior to the index event, any vascular disease (coronary/periphery/carotid artery disease) or a history of heart failure. The mean follow‐up time was 20 ± 12 months.

### 3.4 Study design and trial flow chart

**FIGURE 1:**
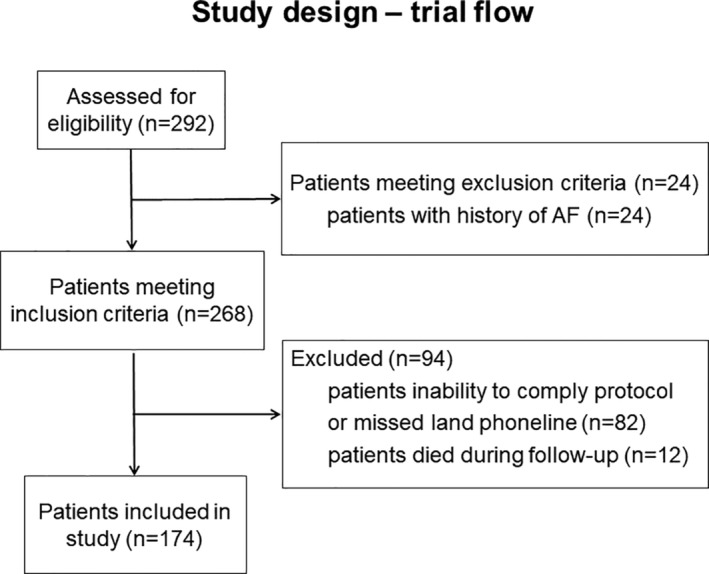
In 3 years (01/2014–11/2016), 292 patients presented at our department of ophthalmology; 24 patients met exclusion criteria. Of the 268 patients meeting inclusion criteria, 94 patients were excluded (82 patients were unable to comply study protocol or missed land phone line; 12 patients died in follow‐up of 20 ± 12 months). Overall, 174 patients could be included. AF, atrial fibrillation

Between 01/2014 and 11/2016, 292 patients with a (C)RAO event were admitted at our ophthalmology department (Figure 1). Of these, 268 patients met the inclusion criteria; 24 patients were excluded based on an existing history of AF. Furthermore 94 patients were excluded due to different reasons, for example, inability to comply with the protocol, missing land phoneline (*n* = 82) or death during follow‐up (*n* = 12).

### 3.5 Statistical analysis

Continuous variables were presented as mean ± standard deviation, categorical variables as numbers and percentages. Normally distributed data were compared using the unpaired Student's *t* test and non‐parametric variables using the Mann–Whitney‐U test, respectively. All statistical analyses were performed using the SPSS software (version 20.0; SPSS Institute, Chicago, IL); a *p*‐value <0.05 was considered statistically significant.

### 3.6 Ethics

All examinations and follow‐up analyses were according to the Declaration of Helsinki and were approved by the local ethics committee (Protocol number 17–079).

## 4 RESULTS

### 4.1 Patient characteristics

Evaluation of demographic variables of all included patients (*n* = 174, see Table 1) revealed a mean age of 74.2 years. 59.2% of patients were ≥ 75 years old. About 60% were male and in half of the cases (49.4%) the occlusion was related to the central retinal artery.

**TABLE 1:** Patients characteristics and comorbidity

Patient characteristics/comorbidity (=number of patients)	Patients included in study
	*n* = 174
Male, *n* (%)	107 (61.5%)
Age, years (mean ± SD)	74.2 ± 13.0
CRAO, *n* (%)	86 (49.4%)
CHA_2_DS_2_VASc‐Score equivalent, excl. (C)RAO (mean ± SD)	3.7 ± 1.7
Low risk (score 0) *n* (%)	5 (2.9%)
Intermediate risk (score 1–2) *n* (%)	37 (21.3%)
High risk^2^, ^12^, ^13^, ^17^, ^22^, ^23^, ^24^, ^25^ *n* (%)	132 (75.9%)
CHA_2_DS_2_VASc‐Score equivalent, incl. (C)RAO (mean ± SD)	5.3 ± 1.4
low risk (score 0) *n* (%)	0 (0%)
intermediate risk (score 1–2) *n* (%)	6 (3.4%)
high risk^2^, ^12^, ^13^, ^17^, ^22^, ^23^, ^24^, ^25^ *n* (%)	168 (96.6%)
Heart failure, *n* (%)	27 (15.5%)
Arterial hypertension, *n* (%)	145 (83.3%)
Age 65–74 *n* (%)	32 (18.4%)
Age ≥ 75, *n* (%)	103 (59.2%)
Diabetes mellitus, *n* (%)	34 (19.5%)
Stroke, *n* (%)	27 (15.5%)
Vascular disease, *n* (%)	69 (39.7%)
Coronary artery disease, *n* (%)	39 (22.4%)
Periphery artery disease, *n* (%)	20 (11.5%)
Carotid/vertebral stenosis, *n* (%)	42 (24.1%)

*Note:* Table 1: In the table the patient characteristics as well as cardiovascular risk factors is depicted. To evaluate the potential risk for stroke, for each patient a CHA2DS2‐VASc‐Score equivalent was calculated as if atrial fibrillation would be present. We want to distinguish the high mean CHA_2_DS_2_‐VASc‐Score when counting (C)RAO as an stroke equivalent. (C)RAO: (central) retinal artery occlusion.

For comparison reasons, we evaluated an equivalent to the CHA_2_DS_2_‐VASc‐Score despite AF was not diagnosed yet. Taking (C)RAO as stroke/TIA equivalent counting for two points, the mean CHA_2_DS_2_‐VASc score was 5.3 ± 1.4; the CHA_2_DS_2_‐VASc score regardless of (C)RAO was 3.7 ± 1.7. The most common risk factor was arterial hypertension 145/174 patients (83.3%) followed by age ≥ 75 years (59.2%). About 40% of patients suffered from a known vascular disease. This was attributable to 24.1% severe carotid disease (NASCET criteria; ≥ 70% stenosis), 22.4% a coronary artery disease and 11.5% a periphery artery disease. About 20% had a diagnosis of diabetes mellitus, while heart failure and stroke in history was documented in 15% each.

### 4.2 Cardiological work‐up

**FIGURE 2:**
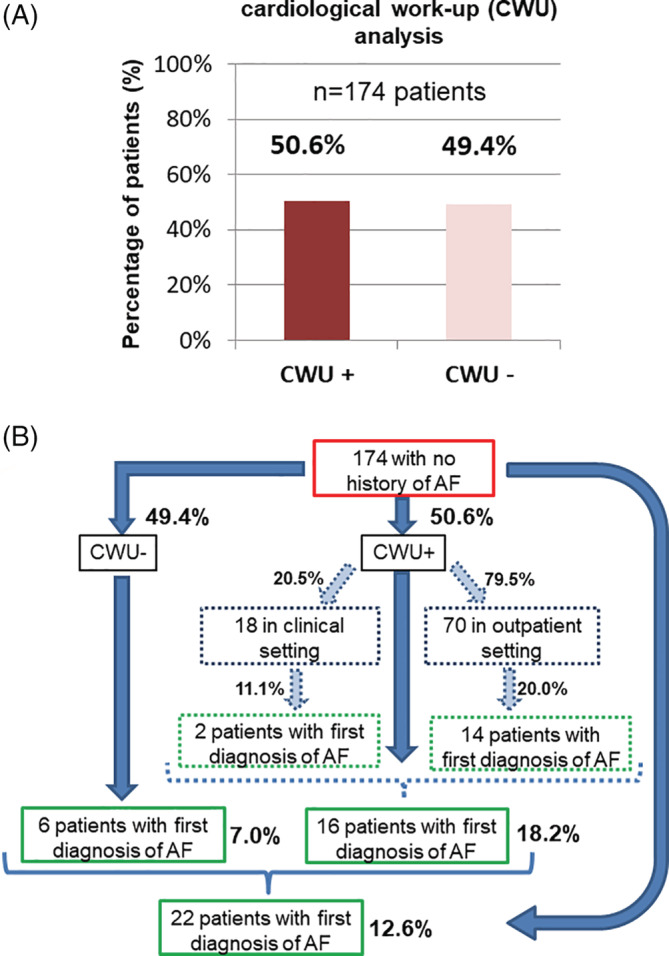
(A) Rate of CWU in the CRAD cohort. Only 50.6% of the studied cohort received a CWU by further ECG. (B) Regarding all included patients (*n* = 174), only 50.6% received a screening for AF with a single 24‐Holter ECG which was realized in 4/5 cases in an ambulatory setting. The number of 1st diagnosis of AF was counted 20% (14/70) in ambulatory setting whereas in clinical setting the number was lower with 11.1% (2/18). So overall we obtained over 18% of new‐onset AF by one single 24‐Holter ECG. At least in 7% AF was documented for the first time in patients who did not receive a specific AF screening. In summary, in 12.6% new‐onset AF could be counted in a FU of 20 months ±12 months. AF, atrial fibrillation

Analysis of primary endpoint performance of a CWU defined as at least one ≥ 24 h‐Holter‐ECG, revealed a CWU rate of 50.6% (CWU+) within the whole study cohort (Figure 2(A)). In most cases (79.5) the screening was performed in an outpatient setting. Figure 2(B) depicts the types of AF detection and further outcomes of CWU+ versus CWU‐ in detail. In the allover cohort, 12.6% of patients showed a new diagnosis of AF within follow‐up period of 20 ± 12 months. In the CWU+ group new‐onset AF was detected in 18.2% by one single 24 h‐Holter‐ECG (*n* = 16/88 cases). Of note, also within the patients receiving no specific subsequent AF screening after the index event (CWU‐ 49.4%), still in 6 patients (7%) AF was diagnosed during follow‐up due to clinical manifestation and further ECG diagnostic.

### 4.3 Subgroup analysis of patients with versus no new‐onset AF

Analysis of demographic variables of patients with de‐novo diagnosed AF versus patients without overt AF occurrence revealed that the presence of heart failure was the only significant different variable between the cohorts (no‐AF 13.8% vs. new‐onset AF 27.3%; *p* < 0.05; see supplemental Table S1).

### 4.4 Neurological work‐up

In a next step, we evaluated the degree of neurological work‐up in all patients in FU. In contrast to CWU, the majority of 86.7% patients (*n* = 151/174) received a duplex ultrasound of carotid arteries (NWU+). In the majority of these patients 140/151 (92.7%) atherosclerotic plaques were observed, whereas 42/151 patients (27.8%) had a significant stenosis ≥ 70% of brain supplying arteries.

### 4.5 Potential cause of (C)RAO

**FIGURE 3:**
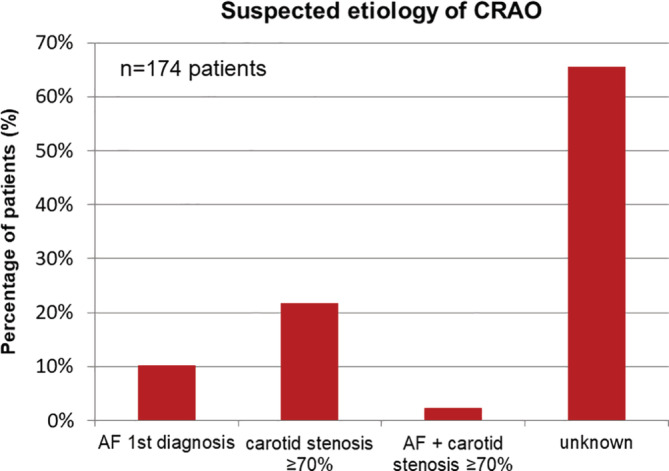
Etiology of (C)RAO: Patients with AF documented for the 1st time had a prevalence of 10.3%. Severe stenosis of carotid arteries (NASCET criteria; ≥70%) were known in 21.8%. In 2.3% patients named both diseases so a definitive attribution is not possible. In most cases – over 65% – no etiology could be identified. (C)RAO: (central) retinal artery occlusion; AF: atrial fibrillation

To evaluate the potential underlying source of the (C)RAO event, we performed an analysis with respect to the two main known causes represented by new‐onset AF or significant carotid disease (NASCET criteria; defined as ≥ 70%). Figure 3 illustrates the distribution of the findings. De‐novo diagnosed AF, which was present in 10.3% (18/174), while ipsilateral significant stenosis (≥ 70%, NASCET criteria) of carotid arteries was nearly twice as often observed in 21.8% of the patients. In a small group (4/174 patients; 2.3%) both entities, AF and carotid disease, could be observed. In most cases (65.6%) none of the two entities could be detected.

### 4.6 Antithrombotic therapy and recurrent ischemic events

**FIGURE 4:**
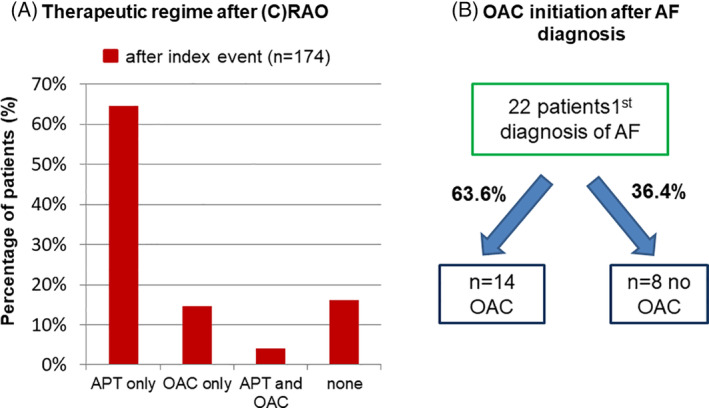
In figure (A), the analysis of blood‐thinning therapies is represented: In most cases (about 70%) APT was taken only. OAC were only named in 8.0% and 3.4% had ASS as well as OAC, remaining 18.4% taking none. Figure (B) shows that patients with 1st diagnosis of AF (*n* = 22) did not take a OAC in more than 36%. Thus only 2/3 of patients with AF had a secondary prophylaxis against stroke. AF, atrial fibrillation; APT, anti‐platelet therapy; (C)RAO, (central) retinal artery occlusion; OAC, oral anticoagulation

Further we analyzed antithrombotic treatment after the index event. About 70% of the patients were treated with anti‐platelet therapy whereas 8.0% received an OAC. Both anti‐platelet therapy as well as OAC was noted in 3.4%, while no specific therapy was present in 18.4% of the patients (Figure 4(A)). Figure 4(B) depicts the subgroup of patients with established diagnosis of AF and with respect to the type of secondary prevention. Our retrospective analysis showed that patients with new‐onset AF were in consequence in 64% treated with OAC, while 36% did actually not receive any guideline recommended therapy for prevention of further thromboembolic events (TE) despite the diagnosis of AF on the past.

In addition to the prevention therapy, we investigated recurrent embolic events after index event. During follow‐up 23/174 patients suffered from a stroke or recurrent (C)RAO (see Figure S1). In a sub‐analysis, we aimed to evaluate if patients with new‐onset AF had more recurrent TE than patients without AF. Our analysis revealed that patients with new‐onset AF (*n* = 22) experienced as often as patients without AF a recurrent thromboembolic event (13.6% vs. 13.2%, *p* = 0.95; see Figure S1a).

## 5 DISCUSSION

According to guidelines, acute retinal arterial ischemia is a stroke equivalent and demand equal diagnostic work‐up and at least monitoring over 24 h.^19^ In this study we could show, that in a selected sub‐form of a stroke‐equivalent, (C)RAO, there is a clear lack of a cardiological diagnostic work‐up in the daily routine, which could have potential impact on re‐current events and clinical outcome.

### 5.1 Potential role of AF

The American Heart Association and National Stroke Association recommend in patients with stroke an immediate evaluation in certified stroke centers which include an ECG as soon as possible after the event. Prolonged cardiac monitoring (inpatient telemetry or Holter monitor) should be performed in (C)RAO patients with an unclear etiology after initial brain/vessel imaging and ECG.^23^, ^25^, ^26^ Besides the fact of an insufficient CWU in our studied (C)RAO cohort we aimed to evaluate the rate of new‐onset AF in the setting of a CWU and in case no CWU was performed after the index event.

The CRYSTAL AF trial was the first big trial, which investigated systematically the role of a diagnostic measure with respect to an AF diagnosis after a cryptogenic stroke event.^1^ In this cohort, patients were followed with an implantable loop recorder (ICM) for uninterrupted ECG screening. The intervention group (implantation of ICM) had a rate of 12.4% new‐diagnosed AF after 12 months compared to 2% in the control group. Looking at the absolute numbers, our results seem to be consistent showing AF rates of 18.2% in the CWU group and 7% in the group without CWU over a mean FU of 20 months. However, taken the diagnostic methods, the control group in the CRYSTAL AF, in which at least one 24 h ECG was demanded, reflects much more the CWU group in our trial. This further highlights the hypothesis, that the silent AF rate is much higher in a (C)RAO population compared to a classical cryptogenic stroke or ESUS cohort.

### 5.2 Mode of action

In other studies, severe atherosclerosis of carotid artery seems to be the most common finding with respect to the underlying cause in (C)RAO patients. In the literature the prevalence of significant stenosis of brain supplying arteries differs from 10% to 70% in (C)RAO patients.^12^, ^13^, ^14^, ^15^ In our (C)RAO patients with NWU+, severe carotid artery stenosis (≥NASCET criteria) was diagnosed in 27.8% and fits quite well with previous studies.

### 5.3 Therapeutic consequences on treatment strategy

Taking (C)RAO as an equivalent of stroke, treatment with an antiplatelet agent is recommended, if there is no contraindication. In our retrospective analysis, over 16% of patients did not take any blood‐thinning drug at all which could indicate patients missed an adequate internal assessment after (C)RAO. This hypothesis could also explain – at least in some extend – the high number of untreated AF (1/3 of cases). Severe bleeding events in the past could be a reason for withholding OAC in 2 patients. Still, taking the CHA_2_DS_2_‐VASc score into account, there were 8/15 patients in which no objective reason for withholding OAC despite a diagnosis AF could be found.

As mentioned above, registry studies provide evidence that patients after (C)RAO have a significantly elevated risk for further cerebral events. One retrospective study, in which even after correcting for cardiovascular risk factors, patients with AF and (C)RAO had an increased risk for future ischemic events (stroke/TE/TIA) with a hazard ratio of 1.39 (95%, CI 1.08–1.79).^24^

In our study population, we obtained rates of recurrent thromboembolic event – stroke or recurrent (C)RAO – in more than 13% of patients during a mean FU of 20 months, which is remarkably high. This is comparable with existing studies; in a Korean study patients with retinal artery occlusion had a risk of subsequent stroke of 19.6% during the 3‐year FU.^18^ Interestingly, in our study patients with diagnosed AF had an equivalent rate of recurrent cerebral events compared to patients without the diagnosis of AF. An explanation could be that due to a missing CWU in nearly half of the patients AF was just underdiagnosed in our study cohort.

Taking our results with respect to OAC treatment and recurrent events, these seem to be in part comparable to the results observed in two recently published prospective randomized studies in ESUS patients (RESPECT, NAVIGATE^2^, ^27^;). In patients with cryptogenic stroke or ESUS, therapeutic anticoagulation failed to result in a benefit compared to antiplatelet therapy with aspirin with respect to preventing recurrent events It might be speculated that AF was not the main causal etiology in the study cohort; in NAVIGATE the diagnosis of AF was yield only in 3% in a FU of 11 month.

### 5.4 Potential relevance and clinical implications

This is one of the first studies evaluating the actual numbers of patients receiving a CWU, including a systematic screening for AF as potential source of a systemic thromboembolism after a (C)RAO event. Taken the fact, that (C)RAO is estimated to be caused in >95% by a thromboembolic event, it is noteworthy that in half of cases in our cohort not even a single 24 h‐Holter‐ECG was documented. There is a clear gap between guidelines recommendation and practical performed cardiac assessment as shown in our study cohort. In a Canadian stroke register, the proportion of patients who received at least 24‐hour Holter monitoring within 30 days after stroke/TIA was evaluated. In less than one third of cases, patients with stroke/TIA (n = 17 398) received a guideline‐recommended 24‐hour Holter monitoring, and < 1% prolonged ambulatory ECG monitoring.^28^ These data show that there is a clear underutilization of ECG monitoring in this specific group of patients.

Despite the guidelines, in current practice patients with acute retinal infarction are only rarely evaluated in a similar way as patients presenting with cerebral symptoms. It is known from another study that time to treatment in patients with retinal TIA is much longer than in patients presenting with neurological TIAs (48.5 versus 15.2 days).^29^ In a US study, acute (C)RAO patients were send to an emergency department in 73% when seen by a neurologist and only in 18% when see by a retina specialist.^30^ This data further demonstrates that physicians, especially ophthalmologists, might not be aware that acute retinal ischemia is potentially directly linked to an acute cerebral ischemia and therefore represents a medical emergency. At first, with campaign such as ''ACT FAST'' for public education, patients should be more aware that loss of vision is one of the main symptoms of stroke.^20^ Furthermore, local networks and mutual guideline recommendations among ophthalmologists, neurologists, and cardiologists with stroke expertise should be established aiming to facilitate the fast access from the emergency room into stroke units or hospitalization with adequate medical work‐up.

### 5.5 Perspective

Recent technological developments highlight the possibilities of ECG diagnostic by so‐called wearables.^31^ Taken our results with a high rate of AF diagnosed during FU, it seems justified to discuss, if patients after an (C)RAO event might be a potential target population for wearable devices being capable of ECG diagnostic with respect to AF detection. However, if the subsequent diagnosis of AF and initiation of an OAC will translate in better outcome is completely unclear and has to be addressed by future trials.

### 5.6 Potential limitations

This is retrospective study in a very selected group (C)RAO patients, who initially presented at the ophthalmological department. We did not perform a prospective comparison to patients presenting at first at the neurological department. Based on the retrospective nature there was no uniform standard of CWU performed. Data collection were primary based on patient record files and telephone interviews.

In our study, patients with new‐onset AF experienced a recurrent thromboembolic event as often as patients without AF in the follow‐up, which is quite surprising. On one hand the number of patients is quite small so and the total number of events is a limitation. Furthermore, the mode of follow‐up was based on the retrospective nature of this study, which might lead to an underestimation of clinical events. On the other hand, the duration of follow up was rather short with 20 ± 12 months, which could explain the similar rates of thromboembolic events in both groups.

Still, in perspective of the lack of data, in this special entity our analyses revealed relevant results and can be taken as hypothesis‐generating for future prospective studies.

## 6 CONCLUSION

Our study illustrates that patients with (C)RAO usually presenting to an ophthalmologist at first, although the main cause is potentially a systemic embolic condition. The rate of systematical CWU performed was very low, but rate of AF diagnosis was relatively high. Although these results represent common sense, to the best of our knowledge there is a clear lack of systematic data or previous studies on this topic. Our data highlight a current gap between the diagnosis of (C)RAO and subsequent further diagnostics and therapy, which should be addressed in the future. Implementation of SOPs with recommendation to screen for AF might be required to improve secondary prevention measures in these patients.

## CONFLICT OF INTEREST

Reza Wakili has received consultant fees, speaking honoraria and travel expenses from Biotronik; Boston Scientific and Medtronic; investigator‐initiated funding for research projects (initiated by him) from Bristol‐Myers Squibb/Pfizer and Boston Scientific. Other authors: No conflicts of interest.

## AUTHOR CONTRIBUTION

Nadine Vonderlin and Reza Wakili: Involved in the conduct of the registry and in data acquisition, involved in data analysis and interpretation. Nadine Vonderlin, Karsten Kortüm, Johannes Siebermair, Martin Köhrmann, Tienush Rassaf, Steffen Massberg, Siegfried Priglinger, Stefan Kääb and Reza Wakili: Involved in critically revising the manuscript, have provided final approval, and take full accountability for the work, for all content and editorial decisions.

## Supporting information


**Figure S1** In the background of elevated thromboembolic risk after (C)RAO, we analyzed the number of second cerebral events in FU (20 ± 12 months, Figure 1(A)). The figure shows that 13.2% (23/174) of patients had at least one more embolic event (stroke 7.5%; 2nd (C)RAO 4.0%; stroke and 2nd (C)RAO 1.7%). FU: follow‐up; AF: atrial fibrillation; (C)RAO: (central) retinal artery occlusion.

**Table S1** Patients characteristics and comorbidity in subgroups.

## Data Availability

The data set supporting the results of this article are included within the article and supplementary materials

## References

[1] Sanna T , Diener HC , Passman RS , et al. Cryptogenic stroke and underlying atrial fibrillation. N Engl J Med. 2014;370:2478‐2486. 24963567 10.1056/NEJMoa1313600

[2] Diener HC , Easton JD , Granger CB , et al. Design of Randomized, double‐blind, evaluation in secondary stroke prevention comparing the EfficaCy and safety of the oral thrombin inhibitor dabigatran etexilate vs. acetylsalicylic acid in patients with embolic stroke of undetermined source (RE‐SPECT ESUS). Int J Stroke. 2015;10:1309‐1312. 26420134 10.1111/ijs.12630

[3] Geisler T , Poli S , Meisner C , et al. Apixaban for treatment of embolic stroke of undetermined source (ATTICUS randomized trial): rationale and study design. Int J Stroke. 2017;12:985‐990. 27881833 10.1177/1747493016681019

[4] Hart RG , Sharma M , Mundl H , et al. Rivaroxaban for stroke prevention after embolic stroke of undetermined source. New Engl J Med. 2018;378:2191‐2201. 29766772 10.1056/NEJMoa1802686

[5] Leavitt JA , Larson TA , Hodge DO , Gullerud RE . The incidence of central retinal artery occlusion in Olmsted County, Minnesota. Am J Ophthalmol. 2011;152:820‐823.e822. 21794842 10.1016/j.ajo.2011.05.005 PMC3326414

[6] Smit RL , Baarsma GS , Koudstaal PJ . The source of embolism in amaurosis fugax and retinal artery occlusion. Int Ophthalmol. 1994;18:83‐86. 7814205 10.1007/BF00919244

[7] Yuzurihara D , Iijima H . Visual outcome in central retinal and branch retinal artery occlusion. Jpn J Ophthalmol. 2004;48:490‐492. 15486774 10.1007/s10384-004-0102-y

[8] Feltgen N . Retinaler Arterienverschluss. Ophthalmologe. 2017;2:177‐187. 10.1007/s00347-016-0432-4 28093631

[9] Schmidt D , Schumacher M , Feltgen N . Circadian incidence of non‐inflammatory retinal artery occlusions. Graefes Arch Clin Exp Ophthalmol. 2009;247:491‐494. 18989688 10.1007/s00417-008-0989-y

[10] Uhr JH , Mishra K , Wei C , Wu AY . Awareness and knowledge of emergent ophthalmic disease among patients in an internal medicine clinic. JAMA Ophthalmol. 2016;134:424‐431. 26892039 10.1001/jamaophthalmol.2015.6212

[11] Schumacher M , Schmidt D , Jurklies B , et al. Central retinal artery occlusion: local intra‐arterial fibrinolysis versus conservative treatment, a multicenter randomized trial. Ophthalmology. 2010;117:1367‐1375.e1361. 20609991 10.1016/j.ophtha.2010.03.061

[12] Appen RE , Wray SH , Cogan DG . Central retinal artery occlusion. Am J Ophthalmol. 1975;79:374‐381. 1121994 10.1016/0002-9394(75)90609-1

[13] Douglas DJ , Schuler JJ , Buchbinder D , Dillon BC , Flanigan DP . The association of central retinal artery occlusion and extracranial carotid artery disease. Ann Surg. 1988;208:85‐90. 3389947 10.1097/00000658-198807000-00012 PMC1493569

[14] Hayreh SS , Podhajsky PA , Zimmerman MB . Retinal artery occlusion: associated systemic and ophthalmic abnormalities. Ophthalmology. 2009;116:1928‐1936. 19577305 10.1016/j.ophtha.2009.03.006 PMC2757505

[15] Tomsak RL , Hanson M , Gutman FA . Carotid‐artery disease and retinal‐artery occlusion. Lancet. 1979;1:1084. 10.1016/s0140-6736(79)92979-9 86804

[16] Yen JC , Lin HL , Hsu CA , Li YC , Hsu MH . Atrial fibrillation and coronary artery disease as risk factors of retinal artery occlusion: a Nationwide population‐based study. Biomed Res Int. 2015;2015:374616. 26558268 10.1155/2015/374616 PMC4628970

[17] Chang YS , Jan RL , Weng SF , et al. Retinal artery occlusion and the 3‐year risk of stroke in Taiwan: a nationwide population‐based study. Am J Ophthalmol. 2012;154:645‐652.e641. 22809785 10.1016/j.ajo.2012.03.046

[18] Rim TH , Han J , Choi YS , et al. Retinal artery occlusion and the risk of stroke development: twelve‐year Nationwide cohort study. Stroke. 2016;47:376‐382. 26742801 10.1161/STROKEAHA.115.010828

[19] Olsen TW , Pulido JS , Folk JC , Hyman L , Flaxel CJ , Adelman RA . Retinal and ophthalmic artery occlusions preferred practice pattern. Ophthalmology. 2017;124:P120‐p143. 27742458 10.1016/j.ophtha.2016.09.024

[20] Lawlor M , Perry R , Plant GT . Is the 'Act FAST' stroke campaign lobeist? The implications of including symptoms of occipital lobe and eye stroke in public education campaigns. J Neurol Neurosurg Psychiatry. 2015;86:818‐820. 25385853 10.1136/jnnp-2014-308812 PMC4483785

[21] Hindricks G , Potpara T , Dagres N , et al. 2020 ESC guidelines for the diagnosis and management of atrial fibrillation developed in collaboration with the European Association of Cardio‐Thoracic Surgery (EACTS). Eur Heart J. 2020;42:373. 10.1093/eurheartj/ehaa612 32860505

[22] Anderson DC , Kappelle LJ , Eliasziw M , Babikian VL , Pearce LA , Barnett HJ . Occurrence of hemispheric and retinal ischemia in atrial fibrillation compared with carotid stenosis. Stroke. 2002;33:1963‐1967. 12154246 10.1161/01.str.0000023445.20454.a8

[23] Biousse V , Nahab F , Newman NJ . Management of Acute Retinal Ischemia: follow the guidelines! Ophthalmology. 2018;125:1597‐1607. 29716787 10.1016/j.ophtha.2018.03.054

[24] Christiansen CB , Lip GY , Lamberts M , Gislason G , Torp‐Pedersen C , Olesen JB . Retinal vein and artery occlusions: a risk factor for stroke in atrial fibrillation. J Thromb Haemostasis. 2013;11:1485‐1492. 23663383 10.1111/jth.12297

[25] Easton JD , Saver JL , Albers GW , et al. Definition and evaluation of transient ischemic attack. Stroke. 2009;40:2276‐2293. 19423857 10.1161/STROKEAHA.108.192218

[26] Johnston SC , Albers GW , Gorelick PB , et al. National Stroke Association recommendations for systems of care for transient ischemic attack. Ann Neurol. 2011;69:872‐877. 21391236 10.1002/ana.22332

[27] Rubin EJ . Changing principals, keeping principles. N Engl J Med. 2019;381:1069‐1070. 31509679 10.1056/NEJMe1911103

[28] Edwards JD , Kapral MK , Fang J , Saposnik G , Gladstone DJ . Underutilization of ambulatory ECG monitoring after stroke and transient ischemic attack. Stroke. 2016;47:1982‐1989. 27406109 10.1161/STROKEAHA.115.012195

[29] Streifler JY , Eliasziw M , Benavente OR , et al. The risk of stroke in patients with first‐ever retinal vs hemispheric transient ischemic attacks and high‐grade carotid stenosis. North American Symptomatic Carotid Endarterectomy Trial. Arch Neurol. 1995;52:246‐249. 7872876 10.1001/archneur.1995.00540270034016

[30] Abel AS , Suresh S , Hussein HM , Carpenter AF , Montezuma SR , Lee MS . Practice patterns after acute embolic retinal artery occlusion. Asia Pac J Ophthalmol. 2017;6:37‐39. 10.22608/APO.201690 28161924

[31] Turakhia MP , Desai M , Hedlin H , et al. Rationale and design of a large‐scale, app‐based study to identify cardiac arrhythmias using a smartwatch: the apple heart study. Am Heart J. 2019;207:66‐75. 30392584 10.1016/j.ahj.2018.09.002 PMC8099048

