# Supplementary figures and images for: Central retinal artery occlusion as a first sign of atrial fibrillation: A 3‐year retrospective single‐center analysis
Source: Clin Cardiol. 2021 Oct 28;44(12):1654–61. doi: 10.1002/clc.23673 (PMC8715400; doi:10.1002/clc.23673)

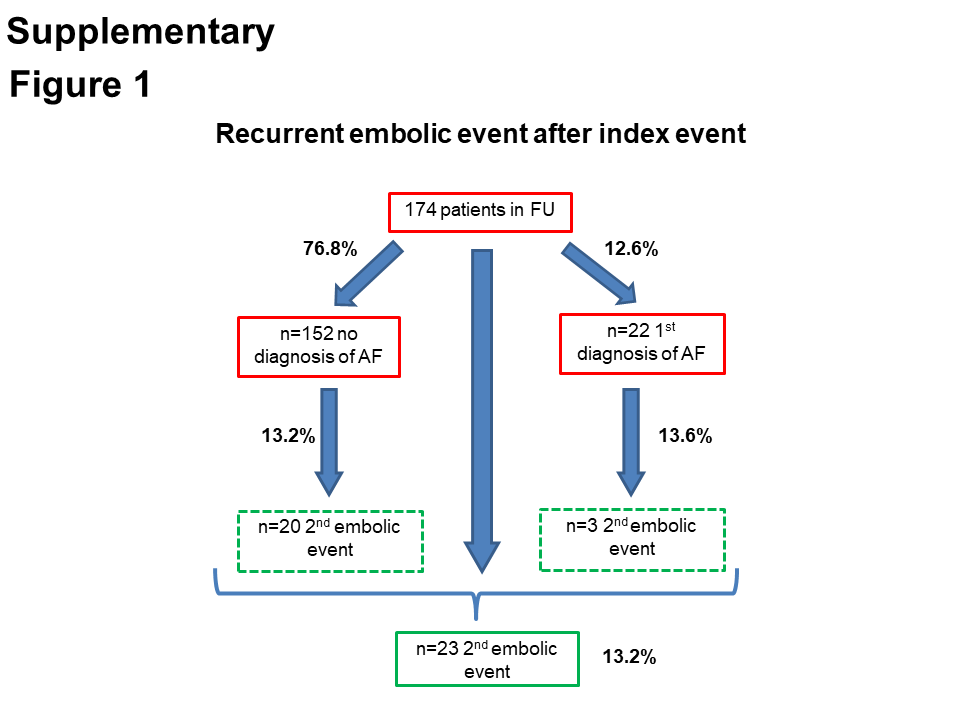

Supplement: Supplementary file 1 — Figure S1 In the background of elevated thromboembolic risk after (C)RAO, we analyzed the number of second cerebral events in FU (20 ± 12 months, Figure 1(A)). The figure shows that 13.2% (23/174) of patients had at least one more embolic event (stroke 7.5%; 2nd (C)RAO 4.0%; stroke and 2nd (C)RAO 1.7%). FU: follow‐up; AF: atrial fibrillation; (C)RAO: (central) retinal artery occlusion. [file CLC-44-1654-s001.tif]
