# Supplementary material for: Central retinal artery occlusion as a first sign of atrial fibrillation: A 3‐year retrospective single‐center analysis
Source: Clin Cardiol. 2021 Oct 28;44(12):1654–61. doi: 10.1002/clc.23673 (PMC8715400; doi:10.1002/clc.23673)
Supplement: Supplementary file 2 — Table S1 Patients characteristics and comorbidity in subgroups. [file CLC-44-1654-s002.docx]

**Supplementary:**

*Figure 1: In the background of elevated thromboembolic risk after (C)RAO, we analyzed the number of second cerebral events in FU (20 ± 12months, figure 1a). The figure shows that 13.2% (23/174) of patients had at least one more embolic event (stroke 7.5%; 2^nd^ (C)RAO 4.0%; stroke and 2^nd^ (C)RAO 1.7%). FU: follow-up; AF: atrial fibrillation; (C)RAO: (central) retinal artery occlusion.*

**Table 1: Patients characteristics and comorbidity in subgroups**

| **Patient characteristics/comorbidity**  (=number of patients) | **Patients included in study** | **Patients no AF in FU** | **Patients 1^st^ diagnosis of AF in FU** | **P value** |
| --- | --- | --- | --- | --- |
|  | n=174 | n=152 | n=22 |  |
| Male, n (%) | 107 (61.5%) | 90 (59.2%) | 17 (77.3%) | p=0.10 |
| Age, years (mean ± SD) | 74.2 ± 13.0 | 73.8 ± 13.2 | 77.0 ± 11.3 | p=0.25 |
| CRAO, n (%) | 86 (49.4%) | 74 (48.7%) | 12 (54.5%) | p=0.61 |
| CHA_2_DS_2_VASc-Score equivalent, excl. (C)RAO (mean ± SD) | 3.7 ± 1.7 | 3.7 ± 1.8 | 3.9 ± 1.2 | p=0.14 |
| low risk (score 0) n (%) | 5 (2.9%) | 5 (3.3%) | 0 (0%) | p=0.39 |
| intermediate risk (score 1-2) n (%) | 37 (21.3%) | 34 (22.4%) | 3 (13.6%) | p=0.35 |
| high risk (2-9) n (%) | 132 (75.9%) | 113 (74.3%) | 19 (86.4%) | p=0.22 |
| CHA_2_DS_2_VASc-Score equivalent, incl. (C)RAO (mean ± SD) | 5.3± 1.4 | 5.3 ± 1.5 | 5.5 ± 1.1 | p=0.17 |
| low risk (score 0) n (%) | 0 (0%) | 0 (0%) | 0 (0%) | n |
| intermediate risk (score 1-2) n (%) | 6 (3.4%) | 6 (3.9%) | 0 (0%) | p=0.34 |
| high risk (2-9) n (%) | 168 (96.6%) | 146 (96.1%) | 22 (100%) | p=0.34 |
| Heart failure, n (%) | 27 (15.5%) | 21 (13.8%) * | 6 (27.3%) * | p=0.001 |
| Arterial hypertension, n (%) | 145 (83.3%) | 126 (82.9%) | 19 (86.4%) | p=0.68 |
| Age 65-74 n (%) | 32 (18.4%) | 29 (19.1%) | 3 (13.6%) | p=0.54 |
| Age ≥75, n (%) | 103 (59.2%) | 87 (57.2%) | 16 (72.7%) | p=0.17 |
| Diabetes mellitus, n (%) | 34 (19.5%) | 30 (19.7%) | 4 (18.2%) | p=0.86 |
| Stroke, n (%) | 27 (15.5%) | 23 (15.1%) | 4 (18.2%) | p=0.71 |
| Vascular disease, n (%) | 69 (39.7%) | 61 (40.1%) | 8 (36.4%) | p=0.74 |
| Coronary artery disease, n (%) | 39 (22.4%) | 32 (21.1%) | 7 (31.8%) | p=0.26 |
| Periphery artery disease, n (%) | 20 (11.5%) | 17 (11.2%) | 3 (13.6%) | p=0.74 |
| Carotid/vertebral stenosis, n (%) | 42 (24.1%) | 38 (25.0%) | 4 (18.2%) | p=0.49 |

*Table 1: In the table the patient characteristics as well as cardiovascular risk factors is depicted. To evaluate the potential risk for stroke, for each patient a CHA2DS2-VASc-Score equivalent was calculated as if atrial fibrillation would be present. We want to distinguish the high mean CHA_2_DS_2_-VASc-Score equivalent* *when counting (C)RAO as a stroke equivalent. Comparing patients with new-onset AF and patients without any history of AF, only heart failure (*) shows a significant difference which could indicate that AF have advanced cases. AF: atrial fibrillation; (C)RAO: (central) retinal artery occlusion.*
